# Tactile stimulation designs adapted to clinical settings result in reliable fMRI-based somatosensory digit maps

**DOI:** 10.1186/s12868-024-00892-x

**Published:** 2024-10-01

**Authors:** Till Steinbach, Judith Eck, Inge Timmers, Emma E. Biggs, Rainer Goebel, Renate Schweizer, Amanda L. Kaas

**Affiliations:** 1 Department of Cognitive Neuroscience, Maastricht University, Oxfordlaan 55, 6228 EV Maastricht, The Netherlands; 2 Brain Innovation B.V., Maastricht, The Netherlands; 3 Department of Medical and Clinical Psychology, Tilburg University, Tilburg, the Netherlands; 4 Functional Imaging Laboratory, German Primate Center, Göttingen, Germany; 5 Leibniz ScienceCampus Primate Cognition, Göttingen, Germany

**Keywords:** human, somatosensory cortex, retest reliability, translational, stroke, upper-limb

## Abstract

Movement constraints in stroke survivors are often accompanied by additional impairments in related somatosensory perception. A complex interplay between the primary somatosensory and motor cortices is essential for adequate and precise movements. This necessitates investigating the role of the primary somatosensory cortex in movement deficits of stroke survivors. The first step towards this goal could be a fast and reliable functional Magnetic Resonance Imaging (fMRI)-based mapping of the somatosensory cortex applicable for clinical settings. Here, we compare two 3 T fMRI-based somatosensory digit mapping techniques adapted for clinical usage in seven neurotypical volunteers and two sessions, to assess their validity and retest-reliability. Both, the traveling wave and the blocked design approach resulted in complete digit maps in both sessions of all participants, showing the expected layout. Similarly, no evidence for differences in the volume of activation, nor the activation overlap between neighboring activations could be detected, indicating the general feasibility of the clinical adaptation and their validity. Retest-reliability, indicated by the Dice coefficient, exhibited reasonable values for the spatial correspondence of single digit activations across sessions, but low values for the spatial correspondence of the area of overlap between neighboring digits across sessions. Parameters describing the location of the single digit activations exhibited very high correlations across sessions, while activation volume and overlap only exhibited medium to low correlations. The feasibility and high retest-reliabilities for the parameters describing the location of the single digit activations are promising concerning the implementation into a clinical context to supplement diagnosis and treatment stratification in upper limb stroke patients.

## Introduction

Behavioral assessments of somatosensory functioning in neurological diseases with motor deficits, especially in stroke, are often performed by physicians, physiotherapists, and occupational therapists to predict outcome, assist treatment planning, and review progress of impaired motor functions of the limbs [49, 68]. Their relevance is confirmed by estimations that somatosensory impairments are present in roughly every second stroke patient, with numbers possibly being underestimated due to a lack of application of standardized somatosensory testing procedures [32, 62]. Effort has therefore been made to advocate and to systematically include standardized assessment of somatosensation in stroke related movement impairments not only in diagnostics but also through recovery and treatment [11]. But there has also been a call for descriptions of the changes in the primary somatosensory cortex (SI) after stroke, to foster a more detailed understanding of their relationship to the functional outcomes [62] and to substantiate the enhancement of somatosensory intervention strategies [7, 12].

Movements, planned and implemented in higher-order motor system brain areas and elicited by the cortical motor output area MI, also critically involve interactions with the cortical somatosensory input area SI. The continuous input of movement related somatosensory information from SI to MI is crucial for the efficiency and preciseness of the motor action [13, 65]. Recent functional magnetic resonance imaging (fMRI) based studies could even show that SI (BA3a and BA3b) receives information about the planned motor functions [25], similar to what is conveyed to MI [1], further emphasizing the significance of not only proprioception but also tactile somatosensation for optimal movement control.

As the recovery of movement is a primary clinical concern in movement-impaired stroke patients, predominantly the disturbance of the motor system and resulting movement oriented therapeutic interventions are investigated and documented. Bringing together evidence from animal research as well as clinical investigations has moved the field forward in describing the distinction between the spontaneous neurophysiological recovery in the first months and the subsequent phase of training induced recovery, providing the knowledge for evidence-based and phase adequate therapeutic interventions (for an extensive account see [36]).

These essential insights are now supplemented by clinical investigations focusing on the somatosensory component. Behavioral assessments of somatosensory perception in upper limb impaired stroke patients show significant recovery of somatosensation in the first 3 months post stroke, indicating spontaneous neurophysiological recovery, as also seen for movements. After 6 months the majority of the assessed stroke patients showed more recovery in somatosensation than in movement, even for severe somatosensory impairments at baseline [70]. This high ability for spontaneous recovery after stroke can be attributed to the known general adaptability of SI to changes in peripheral somatosensory input as shown for the cortical SI representations of the digits of the hand. Not only the long-term usage of the fingers does have a measurable impact on the SI digit maps [16, 18, 44], but also short-term changes of somatosensory input, either decreased through immobilization [39, 67] or increased through somatosensory perception training [8, 9, 50]. But the higher percentage of somatosensory recovery in stroke patients does not imply that somatosensation does not have to be taken into account. The assessments also reveal that a full recovery of the somatosensory impairment is a prerequisite for a full movement recovery [70]. Another study in upper limb stroke patients [5] indicates that somatosensory tasks that involve more active motor aspects result in a lower observed recovery, as well as a lower association between potential and observed recovery. So, despite the good recovery of general somatosensation in upper limb stroke patients, the aspects of somatosensation that are directly involved with movements seem to show a different, diminished recovery.

To be able to gain additional insights into these divergences, not only a larger number of detailed clinical behavioral assessments would be needed, but also parallel functional MRI measurements determining the cortical activation in the SI digit map as a first step towards a more comprehensive description of the role of somatosensation in the upper limb movement recovery in stroke. First, clinical attempts have been already achieved, obtaining activation in the SI digit area with an active, single digit button press approach [17, 52]. But the reported information content regarding SI activation can be questioned considering that MI is twice as wide as the adjacent SI, thus yielding a larger fMRI signal, and the usage of voxel sizes larger than the width of SI. This is further complicated by general findings of differences in the location of MI/SI hand/digit maps as well as newly described overarching action maps in sub-millimeter fMRI measurements [28], which were confirmed along the entire MI map [27], revising the classical motor homunculus topography [47].

We thus see the potential of the passive stimulation approach in mapping the SI digit area, providing a supplementary description of the stroke related layout as well as the consequent adaptability of individual digit maps. As a first step towards this goal, we implemented an adaptation of the approaches currently applied in the state-of-the-art 7 T MRI studies into a 3 T clinical MRI setting, with shorter measurement times, larger volume coverage, decreased spatial resolution, and a less complex data analysis, aiming to describe the validity and retest-reliability of the obtained parameters of the S1 single digit maps. Within the clinical adaptation, we test two different established mapping approaches, Travelling Wave (TW) and Blocked Design (BD) [3, 34, 35, 51, 52, 55] for their feasibility in achieving all five single digit activations within Brodmann Area 3b of SI, and compared parameters of localization and extent of single digit activation to assess validity. To explore the retest-reliability we repeated the MRI measurements in a second session approximately 2 weeks apart, for a description of the variation in the location as well as the extent of the single digit activation and its overlap with neighboring digits. Although this study is an extended assessment of a clinically adapted approach it has been conducted in neurotypical volunteers. The long measurements for the comparison of the approaches would have not been reasonable in patients and the description of the retest-reliability would have been compromised by the confounding factors associated with the clinical status. To still reflect the envisioned usage in the clinical context, we not only report the group averages and variance, but also provide the individual values describing the entire range of the data. This envisions the potential clinical situation, with the first single digit map layout being used in diagnostics and the following measurement(s) capturing the spontaneous recovery as well as further improvements with treatments associated with changes in the usage of the hand and fingers.

## Methods and material

### Participants

Seven neurotypical participants (mean age: 24.9 ± 2.1 years; four females, three males) were recruited via word-of-mouth advertisement. All participants were right-handed, with no self-reported injuries or sensory impairments to their hands, and no self-reported history of neurological disorders. Participants received €30 for their participation. The experiment was approved by the local ethics review committee of Maastricht University and participants gave written informed consent about their participation in the experiment.

### Study procedure

In this within-subject design, participants attended two MRI sessions (separated by 14.3 ± 7.4 days; Range: 7–24 days). No specific instructions were given for the time between the two sessions; it was assumed that participants continued their regular activities. Each 2-hour-session consisted of one structural MR acquisition and four functional digit mapping measurements. Eight additional functional MR measurements obtained in each session are not considered in the present analysis. These other measurements included mapping of the non-dominant hand, both hands at the same time, and active finger tapping sequences. Since the focus of the present analysis is on retest reliability, both sessions included the same measurements.

### Mapping procedure

**Fig. 1:**
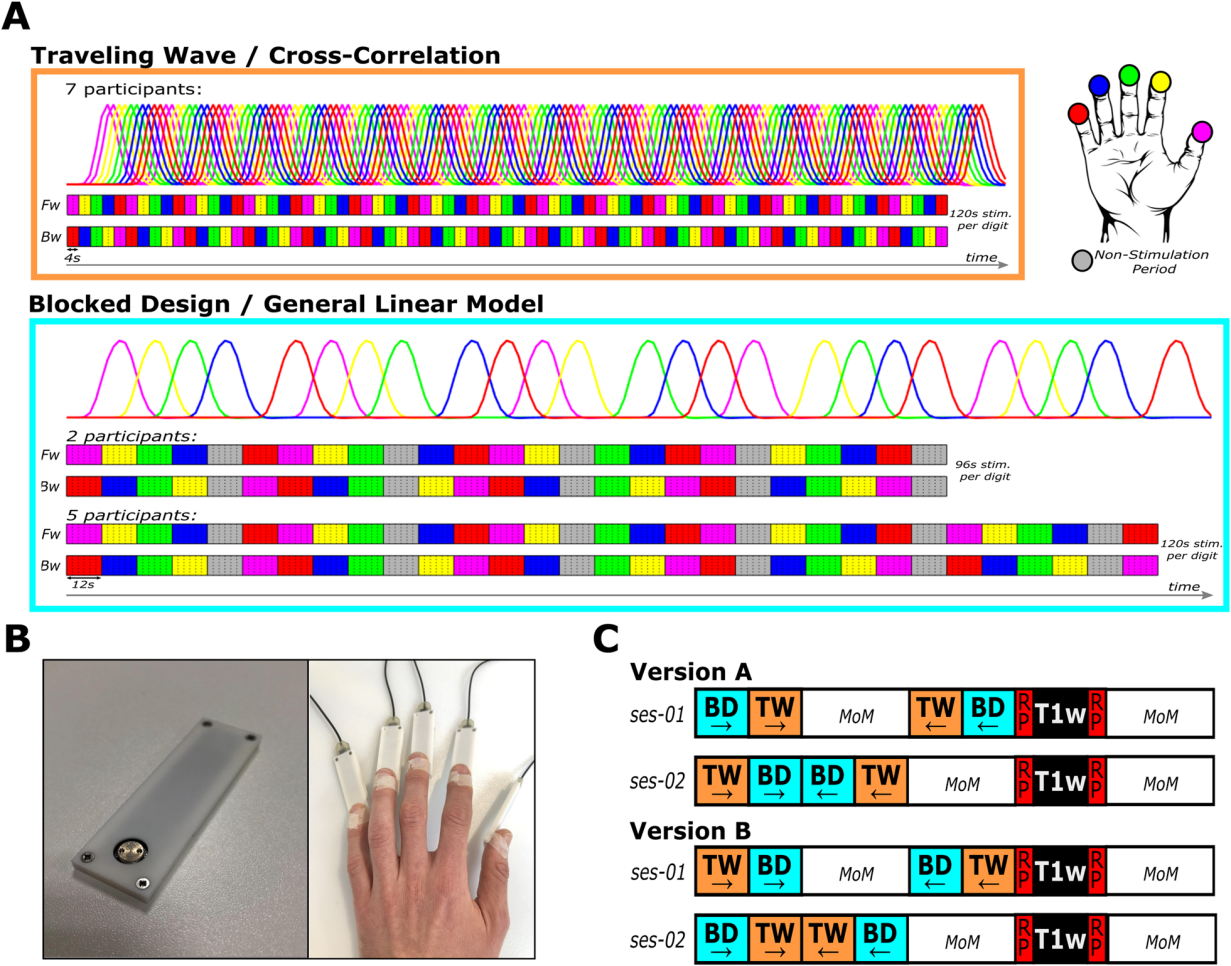
Stimulation device and stimulation designs. **A** Traveling wave (TW) and blocked design (BD) consist of two stimulation directions: Forward (Fw; upper row) with stimulation moving from D1 to D5 and Backward (Bw; lower row) with stimulation moving from D5 to D1. The TW consists of 4 s stimulations of each digit, repeated 15 times in both stimulation directions. The BD consists of 12 s stimulations of each digit and a 12 s non-stimulation rest period (gray) after every fourth digit stimulation. TW was analyzed using cross correlation with two predictors per digit shifted by one TR (2 s). BD was analyzed using a standard GLM analysis with one predictor per digit. **B** Mini piezotactile stimulators (mPTS) with a round, MRI-compatible, metal probe (left panel), delivering a 25 Hz vibrotactile stimulation, were attached to the most distal phalanx of all five digits of the participants right hand (right panel). **C** The order of the stimulation designs within the two sessions was counterbalanced across the participants by assigning them to either Version A or B. In each version, both sessions began with the Fw runs of the two designs, starting with either BD or TW (counterbalanced across sessions). Then, the Bw runs were acquired in the opposite order. During the first session, multiple other measures not relevant for the current experiment (MoM) were acquired between the Fw and Bw runs. In the second session, MoM were acquired after the Bw runs. Lastly, the T1-weighted scans (T1w) were always preceded and followed by functional scans with reversed phase encoding direction (RP), required for EPI distortion correction of the functional scans of interest. All sessions ended with the acquisitions of MoM

During the functional measurements, participants were asked to keep their eyes open and to fixate on a cross back-projected to the center of a screen at the end of the scanner bore. At the same time passive vibrotactile stimulation was applied through five separate modules of a mini piezotactile stimulator (mPTS; Dancer Design, Merseyside, United Kingdom; Fig. 1B) attached to the most distal phalanx of the five digits of the right hand. A stimulation frequency of 25 Hz was delivered through a metal probe (diameter: 6 mm) positioned centrally at the top of the module, moving approximately 0.5 mm up and down. This frequency corresponds to the flutter range, optimally activating the rapidly adapting type 1 afferent fibers [51]. Order and timing of the stimulation was defined by two different stimulation designs, the traveling wave (TW) and the blocked design (BD), as specified below.

#### Traveling wave

During the measurement runs with TW stimulation (Fig. 1A), the five digits of the right hand were repeatedly stimulated in successive anatomical order: thumb (D1), index finger (D2), middle finger (D3), ring finger (D4), little finger (D5). Two different directions of stimulation were acquired in separate measurement runs: forward (D1-D2-D3-D4-D5-D1-D2-D3….) and backward (D5-D4-D3-D2-D1-D5-D4-D3…). For one digit, each stimulation period lasted 4 s, resulting in a TW-cycle of 20 s for all five digits. Each forward or backward TW fMRI measurement run consisted of 15 cycles, resulting in a total acquisition time of 5 min and 20 s, including 10 s non-stimulation periods at the beginning and the end. The combined acquisition time of both TW stimulation directions was 10 min and 40 s, i.e. each digit was stimulated for 120 s across both stimulation runs. The forward and backward stimulation directions were acquired in a counterbalanced fashion (Fig. 1C).

#### Blocked design

The BD (Fig. 1A) was based on the approach used by RS and colleagues [52, 53, 56]. Digits were stimulated for 12 s in anatomical succession (from D1 to D5), but, contrasting the TW approach, with non-stimulation rest periods included after every fourth digit stimulation. This resulted in a long BD cycle in which each digit was stimulated four times and five rest periods were incorporated after every fourth digit stimulation (D1, D2, D3, D4, rest, D5, D1, D2, D3, rest, D4, D5, D1, D2, rest, D3, D4, D5, D1, rest, D2, D3, D4, D5, rest), i.e. once before and once after the stimulation of each of the five digits. Based on the 12 s stimulation periods for each digit stimulation and the non-stimulation periods, one BD-cycle lasted 5 min. The two stimulation directions were acquired in a counterbalanced fashion (Fig. 1C).

The long stimulation of 12 s, as well as the non-stimulation rest periods, are both elements taken from the classical block design [46, 58]. As the stimulation of the single digits occurs in successive anatomical order, the sparse number of rest periods also follows a specific”wandering” pattern of changing position in an orderly manner. Being placed at least once before and after each digit stimulation allows a more balanced general linear model for the analysis of the associated BOLD-activation of each digit.

The BD runs of the first two participants were matched in acquisition time to the TW run of 5 min and 20 s consisting of one BD cycle (5 min) plus a non-stimulation period of 10 s at the start and end, resulting in a total acquisition time of 10 min and 40 s for combined forward and backward stimulation. The total stimulation time per digit was 96 s total compared to 120 s total stimulation time in the TW design.

In the remaining five participants the BD paradigm was lengthened to compensate for the unequal total digit stimulation time. Five 12 s stimulations, one for each digit, plus a non-stimulation rest period after the fourth digit stimulation were added at the end of the runs, resulting in the total duration of stimulation of 120 s for each digit. This increased the acquisition time of the BD to 6 min and 40 s per run (8 s baseline, 5 min BD cycle plus 1 min and 12 s stimulation and a 20 s non-stimulation period after the last digit stimulation) and the total acquisition time to 13 min and 20 s for both stimulation directions.

#### Attentional task

Since attention to touch increases somatosensory cortical activation [31] and counteracts potential habituation effects, short time segments without stimulation were inserted within the period of the vibrotactile stimulation [3, 52–54, 57]. In the TW design, seven 100 ms long interrupts were included in every 4 s digit stimulation spaced by 500 ms. Since the interrupts were rather short and difficult to perceive, participants were asked to count the total number of TW stimulation cycles to assure that their attention was focused on the stimulation.

In the BD, several short non-stimulation periods of 150 ms were included within the 12 s stimulation of each digit. The number of interrupts and their onset within the stimulation period were constant for each digit stimulation, but differed across stimulations of different digits. The stimulation period of D1 and D5 included five interrupts, D2 and D3 six interrupts, and D4 four interrupts. Participants had to count the total number of interrupts in the vibrotactile stimulation during each run of the BD [52–54, 57] and verbally report this number at the end of each run via the intercom used for communication.

### Data acquisition

MRI data were obtained at a Siemens 3 T Prisma Fit system with a 64-channel head coil (Siemens Healthcare, Erlangen, Germany). Anatomical T1-weighted data were acquired with the 3D whole brain coverage Alzheimer’s Disease Neuroimaging Initiative (ADNI) Magnetization Prepared Rapid Gradient Echo (MPRage) sequence (TR = 2300 ms; TE = 2.98 ms; flip angle = 9°; Bandwidth = 240 Hz/Px; FoV = 256 × 256 mm; number of slices = 192; spatial resolution = 1 × 1 × 1 mm^3^). Functional data were acquired using T2*-weighted Gradient Echo Echo Planar Imaging (GE-EPI) (TR = 2000 ms; TE = 30 ms; flip angle = 77°; Bandwidth = 1786 Hz/Px; Multiband Acceleration Factor = 2; GRAPPA Factor = 2; FoV = 200 × 200 mm; number of slices = 64; spatial resolution = 2 × 2 × 2 mm^3^). An additional functional measurement of two volumes with reversed phase encoding direction (posterior to anterior) was recorded before and after the structural measurement (Fig. 1C), to be used for EPI distortion correction during preprocessing [10]. For two participants these reversed phase encoding data were, due to technical difficulties, not available, so no distortion correction could be performed. Careful inspection of the T2* weighted images showed no constraints for the further analysis of the data of these two subjects, since the digit area of the somatosensory cortex being located mid brain is usually only minimally impacted by the distortion.

To minimize head motion as well as to assure participants’ comfort, foam padding was used inside the head coil. To further reduce head motion during data acquisition medical tape was attached from one side of the head coil across the participants’ forehead to the other side of the head coil so that even small head movement resulted in a slight pull on the participant’s skin, providing detailed tactile feedback, in addition to the minimal fixation of the head [37].

### Data analysis

MRI preprocessing and the GLM-analysis of the BD was carried out using BrainVoyager (Version 22.0, Brain Innovation, Maastricht, The Netherlands). The cross-correlational analysis of the TW design as well as the feasibility and reliability measures were performed using the NeuroElf toolbox (Version 1.1) for Matlab (Version 2020a, MathWorks).

#### Data preprocessing

Structural MR images were intensity inhomogeneity corrected, non-linearly transformed into MNI space (ICBM-MNI 152) and for each participant averaged across the two sessions. Functional MRI data were slice-time corrected (cubic spline interpolation), as well as motion corrected and aligned (detection: trilinear; correction: sinc interpolation) to the run and volume that was temporally closest to the anatomical scan during each session. To correct for low-frequency noise, the functional data were high-pass filtered with a cut-off frequency of 0.01 Hz. EPI distortion correction was obtained with the BrainVoyager plugin COPE 1.1 and to preserve the 2 mm isotropic spatial resolution, no extra spatial smoothing was performed.

Coregistration of functional MRI data to within-session structural MRI scans was achieved with boundary-based registration and transformation of functional data into MNI space was performed with sinc interpolation; in the same step, the spatial resolution of the functional data was interpolated to 1 mm isotropic resolution.

#### Definition of the region of interest

The region of interest (ROI) was defined following the procedure described by Valente and colleagues [63], taking inter-individual anatomical differences into account without the need to manually draw ROIs for each participant.

**Fig. 2:**
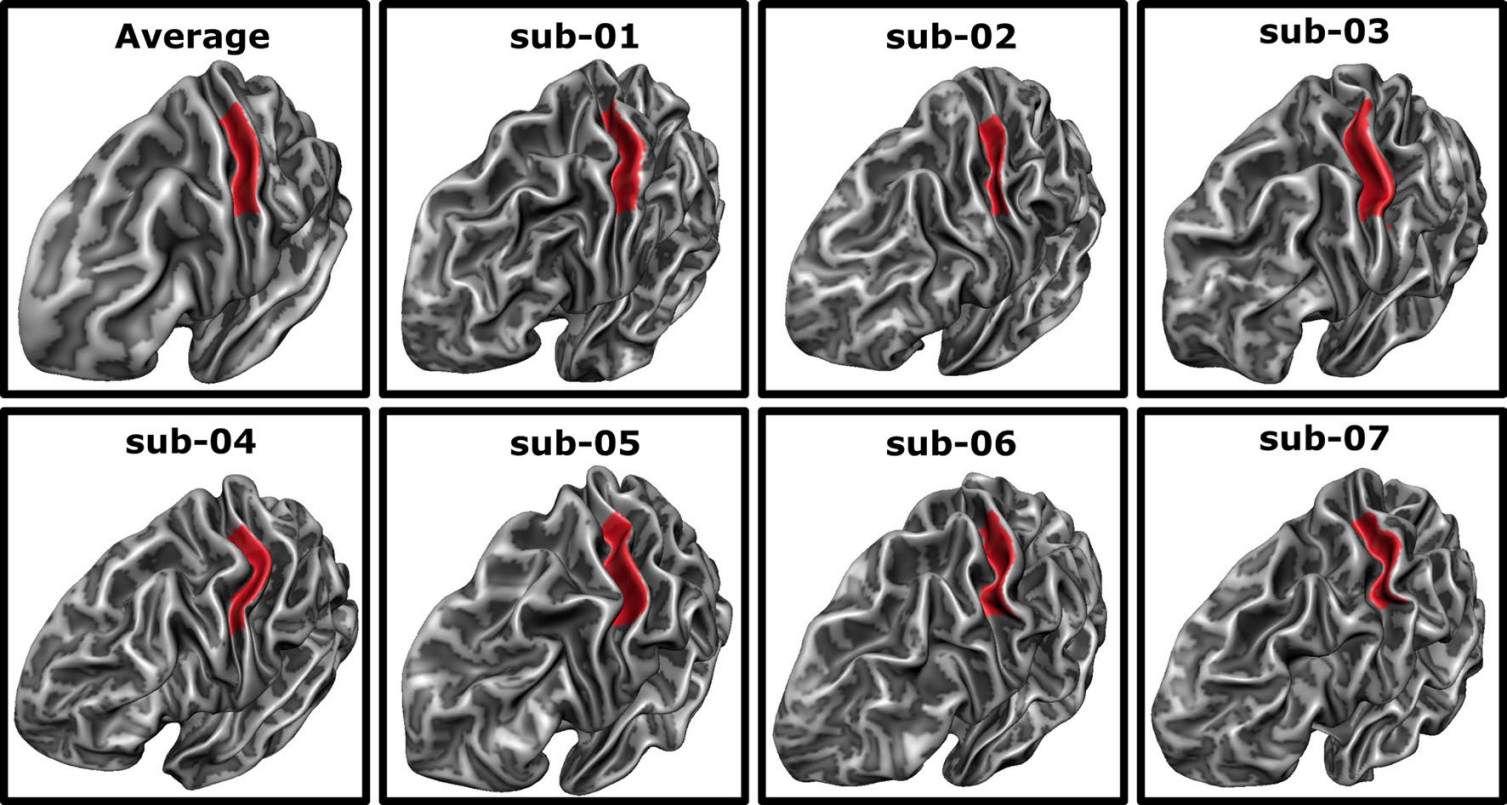
Average and individual regions of interest. The region of interest (ROI) was defined on the averaged surface mesh (top left) as the posterior bank of the central sulcus opposite to the motor hand area. Subsequently, it was backprojected to each participant’s individual surface mesh, covering a similar area in all participants

Individual MNI-normalized cortical surface meshes of the left-hemispheric white–gray matter boundary were created and aligned to a standard sphere using cortex based alignment (CBA) in BrainVoyager [22, 26]. An earlier study has shown a significant and meaningful improvement of the macro-anatomical correspondence of the primary somatosensory hand region between participants after CBA as compared to a volumetric normalization approach [24]. For CBA, a dynamic group average, representing the average curvature information of the specific sample, is created by an iterative alignment of the individual curvature maps from each participant’s hemisphere. This averaged and cortex-based aligned cortical mesh of the seven participants was used to draw a ROI manually, covering the anterior wall of the postcentral gyrus opposite of the hand knob area [69] stretching from the fundus of the central sulcus towards the crown of the postcentral gyrus (see Fig. 2). This ROI is expected to cover the hand area of somatosensory BA 3b as well as parts of BA 3a and BA 1, while reducing the risk of including the large blood vessels over BA 1 [23]. This ROI was subsequently back projected to each participant’s cortical surface mesh using the information from the CBA procedure and in a second step sampled from the surface space to the volume space by expanding it by 2 mm both towards the white matter and the cerebrospinal fluid to assure coverage of the entire gray matter ribbon in that region.

#### First level analyses

##### Cross-correlational analysis of the traveling wave design

The TW was analyzed based on the cross-correlation approach outlined by Kolasinski and colleagues [34, 35] and originally used in retinotopy [19]. A reference predictor was created using a boxcar function including a 4 s “on” period, reflecting the digit stimulation and a 16 s “off” period, reflecting the stimulation of the other digits, which was repeated 15 times to reflect the TW stimulation cycles. This reference predictor was then iteratively shifted 10 times by 2 s, i.e. the TR of the functional measurement, resulting in 10 predictors. To account for the hemodynamic delay each predictor was convolved with a two-gamma canonical response function (Onset = 0,Time to response peak = 6 s; Response dispersion = 1; Response Undershoot ratio: 6; Time to undershoot peak = 16 s; Undershoot dispersion = 1).

Next, the time courses of all voxels within the ROI were correlated with all 10 time-shifted predictors. To allow for statistical tests of the resulting correlation coefficients and correct for their non-normal sampling, Fisher z-transformation was applied [59]. Each of the 10 predictors was then assigned to one of the five fingers, i.e. two predictors to each finger according to the boxcar predictor’s on-period. This approach resulted in two different HRF latencies per digit, i.e., 6 and 8 s (Fig. 1A) [31, 32, 35].

Within each voxel, the Fisher z-transformed correlation values of the two predictors assigned to the same digit were averaged, as were the correlation values from the forward and backward run. This resulted in five correlation values within each voxel, each representing the correlation to one of the five digits. Values in these maps that exceeded a false discovery rate (FDR) corrected threshold (based on all voxels within the ROI) of *q*(FDR) < 0.05 were labeled as active. Individual FDR thresholds are reported in Table 1.

**Table 1:** Individual FDR Thresholds (maxima in parenthesis) for Fisher’s Z based Traveling Wave maps. The number of voxels that were removed based on the functional vein definition is specified in the last column

	D1	D2	D3	D4	D5	Voxels removed
Traveling wave—session 1 [Fisher’s Z-value]						
sub-01	0.21 (0.46)	0.20 (0.47)	0.23 (0.56)	0.19 (0.63)	0.23 (0.46)	0
sub-02	0.19 (0.66)	0.20 (0.70)	0.20 (0.66)	0.18 (0.70)	0.22 (0.40)	1
sub-03	0.17 (1.01)	0.18 (0.85)	0.19 (0.64)	0.18 (0.64)	0.20 (0.54)	5
sub-04	0.27 (0.42)	0.22 (0.48)	0.28 (0.39)	0.23 (0.34)	0.24 (0.47)	0
sub-05	0.20 (0.69)	0.20 (0.87)	0.21 (0.66)	0.19 (0.45)	0.21 (0.46)	0
sub-06	0.18 (0.77)	0.18 (0.87)	0.20 (0.69)	0.18 (0.54)	0.19 (0.72)	0
sub-07	0.21 (0.63)	0.20 (0.65)	0.23 (0.34)	0.19 (0.74)	0.22 (0.49)	0
Traveling wave—session 2 [Fisher’s Z-value]						
sub-01	0.24 (0.39)	0.24 (0.46)	0.23 (0.47)	0.22 (0.53)	0.26 (0.41)	0
sub-02	0.18 (0.79)	0.19 (0.63)	0.19 (0.66)	0.18 (0.76)	0.20 (0.46)	1
sub-03	0.18 (0.71)	0.19 (0.71)	0.21 (0.49)	0.19 (0.50)	0.21 (0.35)	0
sub-04	0.18 (0.65)	0.20 (0.93)	0.20 (0.57)	0.18 (0.50)	0.20 (0.52)	2
sub-05	0.20 (0.74)	0.21 (0.71)	0.21 (0.54)	0.20 (0.42)	0.22 (0.56)	0
sub-06	0.18 (0.70)	0.18 (0.82)	0.19 (0.70)	0.17 (0.72)	0.19 (0.66)	0
sub-07	0.20 (0.64)	0.20 (0.63)	0.22 (0.43)	0.20 (0.64)	0.23 (0.40)	0

A two-way repeated-measures analysis of variance (rm-ANOVA) was performed to investigate systematic differences in FDR thresholds, including the factors SESSION and DIGIT. No significant difference in FDR thresholds between the two sessions could be observed (*F*(1, 6) = 0.2, *p* = 0.671). The five digits differed significantly in their FDR thresholds (*F*(2.1, 12.7) = 21.8, *p* < 0.001), with D3 and D5 exhibiting higher FDR thresholds.

As part of the adaptation to the clinical usage we did not apply the “winner map” approach in which each voxel is exclusively assigned to the digit with the highest statistical value [31, 32, 35]. Utilizing multiple statistical values per voxel enables the analysis of activation overlap between digit activations, facilitating a more thorough investigation of its occurrence and retest reliability. Moreover, this approach permits identifying and excluding voxels that may be influenced by blood vessel activation (see *2.5.3.3. Functional Activation Associated with Veins*).

##### General linear model analysis of the blocked design

For the analysis of the BD, a general linear model (GLM) analysis was applied within the ROI in BrainVoyager. Each digit stimulation was modeled by a boxcar predictor, which was convolved with the same two-gamma canonical response model as used for the TW design (Onset = 0; Time to response peak = 6 s; Response dispersion = 1; Response Undershoot ratio: 6; Time to undershoot peak = 16 s; Undershoot dispersion = 1) (Fig. 1A). A fixed effects analysis including both the forward and backward run was applied in each participant. Each single digit predictor was contrasted against the mean of the predictors of the four remaining digits. This contrast, delimiting the overlap of activation between digits, was introduced to counterbalance the distinguishably larger volumes of activation in the BD design, probably caused by the longer single digit stimulation periods in the BD (12 s) compared to TW (4 s). These larger areas of activation are presumably not reflecting a higher sensitivity to the area of the underlying neuronal digit representation but probably caused by an involvement of larger areas of the vascularity which is not expected to align with the discrete digit layout in BA 3b.

Voxels with statistical values that exceeded an FDR corrected threshold of *q*(FDR) > 0.05 were labeled as active in the somatosensory ROI. Individual FDR thresholds are reported in Table 2.

**Table 2:** Individual FDR Thresholds (maxima in parenthesis) for t-value based Blocked Design maps. The number of voxels that were removed based on the functional vein definition is specified in the last column

	D1	D2	D3	D4	D5	Voxels removed
Blocked design—session 1 [t-value]						
sub-01	2.7 (10.4)	2.8 (13.1)	2.9 (11.1)	2.8 (8.3)	2.7 (10.5)	0
sub-02	2.6 (11.6)	2.8 (9.2)	2.9 (7.4)	2.8 (8.8)	3.0 (9.7)	0
sub-03	2.5 (14.7)	2.8 (12.0)	2.6 (11.8)	2.7 (9.5)	2.5 (7.8)	3
sub-04	2.5 (12.0)	2.7 (14.9)	2.8 (15.5)	2.8 (12.2)	2.5 (7.4)	4
sub-05	2.8 (11.3)	2.8 (14.8)	3.1 (11.0)	3.0 (9.4)	2.9 (8.4)	0
sub-06	2.6 (14.8)	2.6 (17.7)	2.7 (11.8)	2.7 (12.4)	2.6 (13.6)	1
sub-07	2.9 (11.3)	2.8 (11.8)	3.1 (9.0)	2.7 (10.7)	3.1 (5.8)	0
Blocked design—session 2 [t-value]						
sub-01	2.9 (10.1)	2.9 (9.8)	3.0 (9.2)	3.0 (10.6)	2.8 (9.3)	0
sub-02	2.6 (12.2)	2.7 (10.4)	2.9 (9.1)	2.9 (10.4)	2.8 (8.0)	1
sub-03	2.6 (12.5)	2.8 (13.7)	2.6 (7.6)	2.6 (8.0)	2.8 (5.6)	3
sub-04	2.6 (10.0)	2.8 (13.2)	2.9 (12.5)	2.8 (9.7)	2.8 (4.6)	0
sub-05	2.8 (12.0)	2.7 (12.9)	3.0 (7.5)	2.8 (5.4)	3.0 (8.4)	0
sub-06	2.6 (10.7)	2.6 (11.6)	3.0 (11.5)	2.6 (13.7)	2.6 (10.3)	0
sub-07	2.6 (11.3)	2.6 (12.9)	2.9 (7.3)	2.7 (12.2)	2.9 (7.4)	0

A two-way rm-ANOVA with the factors SESSION and DIGIT was applied on the FDR thresholds of the BD. No significant differences between the FDR thresholds of the two sessions could be detected (*F*(1, 6) = 0.2, *p* = 0.637). There was a significant difference between the FDR thresholds of different digits (*F*(2.5, 14.9) = 6.0, *p* = 0.009).

##### Functional activation associated with veins

To control for the influence of draining veins on the BOLD signal, single voxels that showed significant activity for three or more digits [57] were excluded from further analysis, both for TW and BD (Tables 1 and 2. This was done separately per session and design; thus, a single voxel that was excluded in one session or design was not necessarily excluded in the other session or design. The rationale behind this is that in a normal clinical setting, data from only one design and one session will be available and therefore should be sufficient to exclude all potential draining vein artifacts.

##### Visualization of digit maps

To visually inspect the somatosensory activation maps that were elicited by the passive vibrotactile stimulation of the participants’ digits, the data of each session and design were projected to the flattened cortical surface representation of each participant’s left hemisphere. The functional data were projected onto the flattened surface using trilinear interpolation. Data were sampled from a 4 mm ribbon around the reconstructed white–gray matter boundary covering approximately 1 mm into the white and 3 mm into the gray matter direction, using only the maximum value in this range. This was done solely for the purpose of visualization. All analyses were performed in volume space, except for the calculation of the geodesic distance measure, which were obtained from the vertices of the folded surface map.

##### Identification of single digit activation clusters

The activation of each digit in each design and session was defined as its largest cluster of significant BOLD-activation in volume space that contained the peak voxel (i.e., the voxel with the highest statistical value) within the ROI. In case the largest activation cluster did not contain the peak voxel, either the largest cluster or the cluster containing the peak voxel was chosen, depending on which cluster was closest to the clusters chosen for the neighboring digits.

#### Parameterization of digit activation clusters

Activation clusters were parameterized for comparison across the two designs (validity) and across the two sessions (reliability) using ‘center of gravity’ and ‘D1-D5 distance’ as parameters for the location of the activation as well as ‘volume of activation’ and ‘activation overlap between neighboring digits’ as parameters associated with the volume of activation.

##### Center of gravity

The center of gravity (CoG) was defined as the weighted center of activation of that digit’s activation cluster. The coordinates in volume space of the CoG were determined based on the cluster’s average coordinates weighted by each voxel’s statistical value [20]:

$${CoG}_{j}=\frac{{\sum }_{i=1}^{{n}_{j}}{t}_{i}{x}_{i}}{{\sum }_{i=1}^{{n}_{j}}{t}_{i}}$$

with *x* being the coordinate of voxel *i* in cluster *j* with size *n* and *t* being the statistical value of that voxel.

##### D1—D5 distance

The coordinates of the CoGs of D1 and D5 of each session and each design were projected onto the folded surface mesh of each participant and the geodesic distance, i.e., the shortest path along the surface between the vertex representing the D1 CoG and the vertex representing the D5 CoG was obtained using Dijkstra’s algorithm [15].

Even though the distance between D1 and D5 is a measure to describe the size of the cortical digit representation it is included as a location-based parameter, as it critically depends on the localization of the single digits’ activation.

##### Volume of activation

The volume of activation of the single digit representation was defined as the volume of the digit’s activation cluster within the anatomically predefined SI ROI, calculated in mm^3^.

##### Overlap of activation

The overlap of the activation clusters of two anatomically neighboring digits (D1 + D2, D2 + D3, D3 + D4, and D4 + D5) was calculated using the Dice Coefficient (DC) [14]:

$$Dice_{A, B} = \frac{{2 \times \left| {A \cap B} \right|}}{\left| A \right| + \left| B \right|}$$

where |A ∩ B| represents the activation volume shared between the two clusters A and B and |A| and |B| represent the activation volumes of the clusters A and B, respectively. It describes the volume of activation that is overlapping for the two digits relative to the additive volume activated for the two digits. The DC ranges between 0 and 1, with higher values indicating more overlap between the activation of the two digits.

#### Validity: statistical testing

To quantify the validity of mapping the somatosensory digit area using the two adapted mapping procedures, we statistically compared the four extracted map parameters of the first session across the two designs. We expect that both mapping designs produce similar results, similar to those reported in existing literature, and that they likely reflect the main aspects of the underlying somatosensory digit map.

The three coordinate values of the CoG were compared with three two factorial repeated measures (rm)-ANOVAs including the factors DESIGN (TW and BD) and DIGIT (D1 – D5).

The D1-D5 distance was compared across designs using a paired sample t-test.

The volumes of activation were compared with a two factorial rm-ANOVA including the factors DESIGN (TW and BD) and DIGIT (D1 – D5).

The overlap of activation of neighboring digits were compared with a two factorial rm-ANOVA with the factors DESIGN (TW and BD) and DIGITPAIR (D1 + D2, D2 + D3, D3 + D4, D4 + D5).

For all rm-ANOVAs the normality assumption was checked using the Shapiro–Wilk test (Table 3) and homoscedasticity was acknowledged by considering the Greenhouse–Geisser corrected statistical values.

**Table 3:** Tests of normality. Results of the Shapiro–Wilk tests for all data included into the rm-ANOVAs

	Traveling wave			Blocked design		
	*W*	*df*	*p*	*W*	*df*	*p*
Center of gravity—X-coordinate						
D1	0.888	7	0.263	0.953	7	0.760
D2	0.934	7	0.589	0.890	7	0.277
D3	0.866	7	0.173	0.890	7	0.276
D4	0.948	7	0.709	0.828	7	0.077
D5	**0.799**	**7**	**0.040**	0.944	7	0.672
Center of gravity—Y-coordinate						
D1	0.818	7	0.062	**0.742**	**7**	**0.011**
D2	**0.750**	**7**	**0.013**	**0.801**	**7**	**0.042**
D3	0.840	7	0.099	**0.783**	**7**	**0.028**
D4	**0.794**	**7**	**0.035**	**0.790**	**7**	**0.033**
D5	**0.589**	**7**	** < 0.001**	**0.754**	**7**	**0.014**
Center of gravity—Z-coordinate						
D1	0.958	7	0.798	0.931	7	0.563
D2	0.897	7	0.315	0.896	7	0.309
D3	0.870	7	0.186	0.850	7	0.124
D4	0.900	7	0.334	0.856	7	0.138
D5	0.887	7	0.261	0.862	7	0.157
D1-D5 distance						
D1-D5	0.931	7	0.564	0.868	7	0.177
Volume of activation						
D1	0.853	7	0.132	0.930	7	0.547
D2	0.892	7	0.284	0.916	7	0.443
D3	0.966	7	0.866	**0.626**	**7**	**0.001**
D4	0.952	7	0.745	0.971	7	0.904
D5	0.877	7	0.214	**0.781**	**7**	**0.026**
Overlap of activation						
D1 + D2	0.953	7	0.755	0.871	7	0.189
D2 + D3	0.984	7	0.975	0.870	7	0.185
D3 + D4	0.968	7	0.884	0.877	7	0.215
D4 + D5	0.885	7	0.247	0.829	7	0.078

Significant test results (i.e., p < 0.05) indicate a non-normal distribution and are highlighted in bold

#### Retest reliability: statistical testing

##### Stability of activation between sessions

To investigate the reliability of the activation pattern of both designs, the DC was utilized to compare the activation patterns across the two sessions by describing two different types of spatial correspondence. Firstly, the spatial correspondence of same single digit activations across the two sessions, with high DC values indicating high retest-reliability of that single digit cluster, both in terms of location and volume, across the two sessions. Secondly, the spatial correspondence of the overlap between neighboring digit activations across sessions. Here, high DC values indicate high retest-reliability of the overlap of neighboring digit activations across the two sessions both in terms of location and volume.

##### Correlational analysis of map parameters

To investigate the retest reliability of the map parameters, they were correlated across the two sessions separately for each mapping design using Pearson correlation. CoG data were correlated separately for each axis (X, Y, and Z), digit, and design, resulting in 30 individual correlation values (3 axes × 5 digits × 2 designs). The D1-D5 distance was correlated separately per design, resulting in two correlation values. The volume of activation was correlated separately for each digit and design, resulting in 10 individual correlation values (5 digits × 2 designs). The overlap of activation was correlated separately for each digit-pair and design, resulting in 8 individual correlation values (4 digit-pairs × 2 designs). Thus, in total, 50 correlation values were calculated and tested whether they were significantly larger than zero (one-tailed test). To correct for the multiplicity problem, FDR correction using a linear step-up procedure was used [2], values that exceeded a threshold of q(FDR) < 0.05 were considered significantly larger than zero. Additionally, for each correlation value, the slope and intercept of the best fitting line was calculated using the least squares method. The best fitting line of a highly reliable design is expected to be close to the line of equality (i.e., 1x + 0).

The individual stability of the CoG locations between the first and second session was described through Euclidean distances. The Euclidean distance was preferred over the surface-based geodesic distance as no transformation to the gray-white matter boundary is necessary, potentially projecting the very small inter-session differences onto the same vertex. Another advantage is the option to relate the inter-session distances between the CoGs to the voxel dimensions of the functional measurement.

## Results

Two somatosensory digit mapping designs, traveling wave (TW) and blocked design (BD), were adapted to clinical conditions and tested for their feasibility, validity, and retest-reliability in seven neurotypical participants. The investigation of feasibility involved examining the completeness of the digit maps for both designs across the two sessions. Validity was assessed by statistically evaluating map parameters that represent the location, volume, and overlap of digit activations derived from the first session. Retest reliability was determined by assessing the stability of these map parameters in both designs over the two sessions through correlation analyses.

### Feasibility

**Fig. 3:**
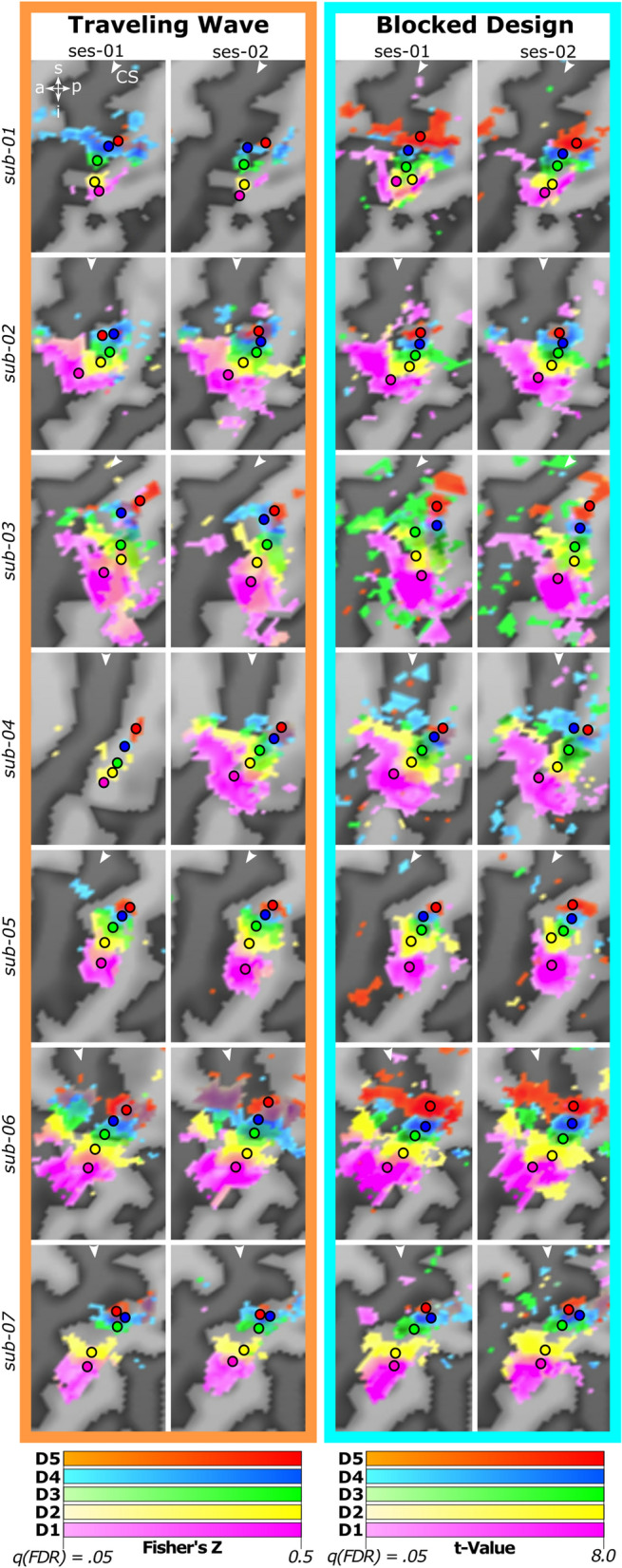
Surface-based single digit activation patterns of all seven participants across designs and sessions. Each row depicts the thresholded statistical map of the BOLD-activation in response to vibrotactile stimulation projected to the flattened brain surface of the individual participant. The left-most dark gray line in each image represents the central sulcus (CS; marked with a white arrowhead) and the light gray line on the right to the CS represents the postcentral gyrus. The top left inset indicates superior (s), posterior (p), inferior (i), anterior (a) directions. Activations of different digits are represented using color gradients in different colors (D1 = magenta; D2 = yellow; D3 = green; D4 = blue; D5 = red). For both Traveling Wave and Blocked Design, the lowest statistical value that is included in the color gradient corresponds to the statistical value at the threshold of q(FDR) < 0.05. The upper limit of the color gradient corresponds to a Fisher’s Z-value of 0.5 for the Traveling Wave maps and to a t-value of 8.0 for the Blocked Design Maps. Vertices above these values are colored in the same color. Overlap of digit activations is displayed as a mixture of colors belonging to the overlapping digits. All seven participants show significant activations in response to stimulation of all five digits along the posterior wall of the CS, creating a topographic digit map. The location of activation as well as the extent of activation is stable across sessions and designs with the exception of two cases (first row and fourth row)

Single digit activation patterns, elicited by the two designs in the two sessions, analyzed and thresholded in volume space, were projected onto the flattened cortical surface reconstruction for easier visualization. BD as well as TW design resulted in significant BOLD-activation associated with the passive tactile stimulation of all five digits in all seven participants (Fig. 3). This illustrates that both designs have enough power to map all digits’ representations in SI under circumstances mimicking a clinical setting, i.e. limiting measurement time as only single runs in each stimulation direction were obtained, without having to compromise on the necessary high spatial resolution of a voxel size of 2 mm isotropic. For all participants, the expected anatomical succession of the digit activations can be seen along the posterior wall of the central sulcus (dark gray) and the shoulder of the postcentral gyrus (light gray). Activations associated with stimulation of D5 are located most superior and of D1 most inferior, with the remaining digits’ representations positioned in anatomical order between these two endpoints. This sequence of digit activations as well as their location is comparable in all participants, across both mapping designs, as well as across sessions. Also, the area of the elicited BOLD-activations is comparable across the two designs and across the two retest sessions, although with two individual exceptions. One case (Fig. 3; first row) exhibits reduced activation during TW compared to BD, in both sessions, but specifically noticeable during the second session. The other case (Fig. 3; fourth row) is displaying a distinctly reduced activation in the TW design in the first session only, so this can be generally attributed neither to the TW design, nor to the first session.

### Validity

#### Location: single digit center of gravity

**Fig. 4:**
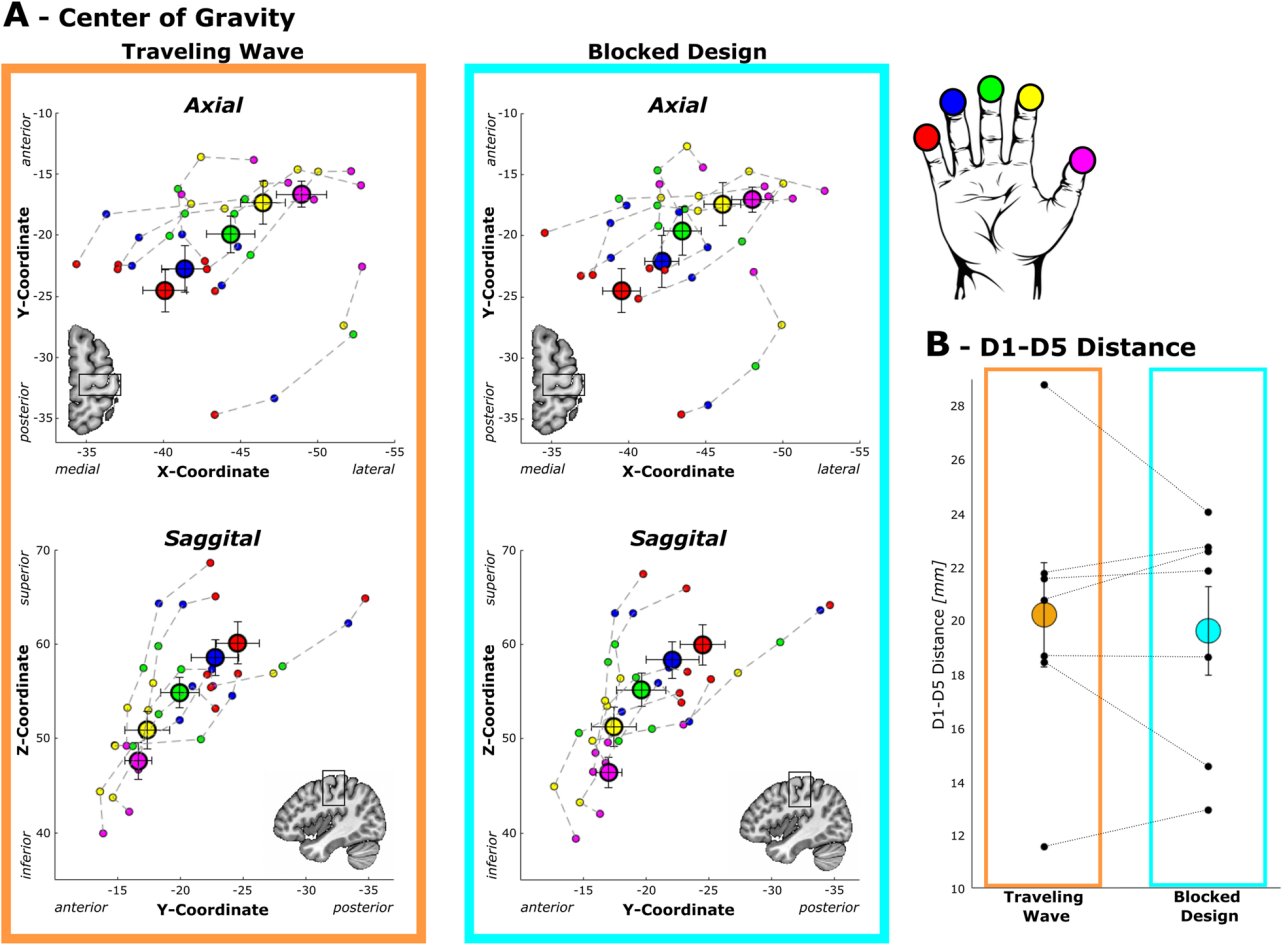
Location based parameter extracted from the digit maps of the first session in MNI volume space. **A** Average locations of each digit’s center of gravity (CoG) in 3D space (large circles) and individual CoGs (smaller circles; each connected succession from D1 to D5 represents one participant). Average and individual CoGs follow the known lateral-to-medial (upper panels), inferior-to-superior (lower panels), and anterior-to-posterior (all panels) succession. No major differences can be observed between the TW and BD. **B** Average (large, colored circles) and individual (small, black circles) geodesic D1-D5 distances for both designs. Connected individual points belong to the same participant. No major differences can be observed between the two designs for the average D1-D5 distances. Error bars represent the standard error of the mean

For a comprehensive description of the location of the BOLD activation, the coordinates of all single digit activation centers of gravity (CoG) of the first session were extracted in 3D volume space (Fig. 4A). The CoGs for TW and BD exhibit the expected digit succession patterns in 3D space across individuals as can be seen in the axial (anterior–posterior and medial–lateral) and sagittal plane (superior-inferior and anterior–posterior). Despite between-subject variations in the position of the succession in 3D space, the group averages of the single digit CoG display the common D1 through D5 succession of distinct digit activation in each coordinate direction. This is confirmed by separate repeated-measures ANOVAs showing a significant effect of the factor DIGIT on all three dimensions: medial–lateral (*F*(1.9, 11.6) = 22.1, *p* < 0.001), anterior–posterior (*F*(1.9, 11.6 = 36.2, *p* < 0.001), and inferior-superior (*F*(2.4, 14.2) = 44.5, *p* < 0.001), all three reflecting the succession of the digit representations along the central sulcus. In all three rm-ANOVAs no significant effects could be obtained neither for the factor DESIGN, nor for the interaction DIGIT x DESIGN. This is also reflected in the small differences in location of the average CoGs of each digit activation between TW and the BD of a Euclidean distance of on average 0.9 mm and a range if 0.5–1.6 mm, which lies within the size of a voxel of 2 mm isotropic. This confirms that both designs result in single digit activation CoGs in very similar locations in 3D space with distinct locations of the single digit CoGs in all three coordinate dimensions.

#### Extent: D1-D5 distance

To describe the extent of the functional digit area we obtained the geodesic distance between the CoGs of D1 and D5 along the brain surface reconstruction. The D1-D5 distance ranging from 11.6 to 28.8 mm, exhibit a large variation in the extent of the somatosensory digit area across subjects (Fig. 4B). The within-subject differences of the D1-D5 distances between the two mapping designs show variation across subjects, but the obtained mean difference of the D1-D5 distances of 1.9 mm (SEM = 0.7 mm) is considerably small. The associated paired samples t-test accordingly shows no significant difference (*t*(6) = 0.61, *p* = 0.566) between the extents of the somatosensory digit areas obtained by the BD and the TW design.

#### Volume of activation: single digit

**Fig. 5:**
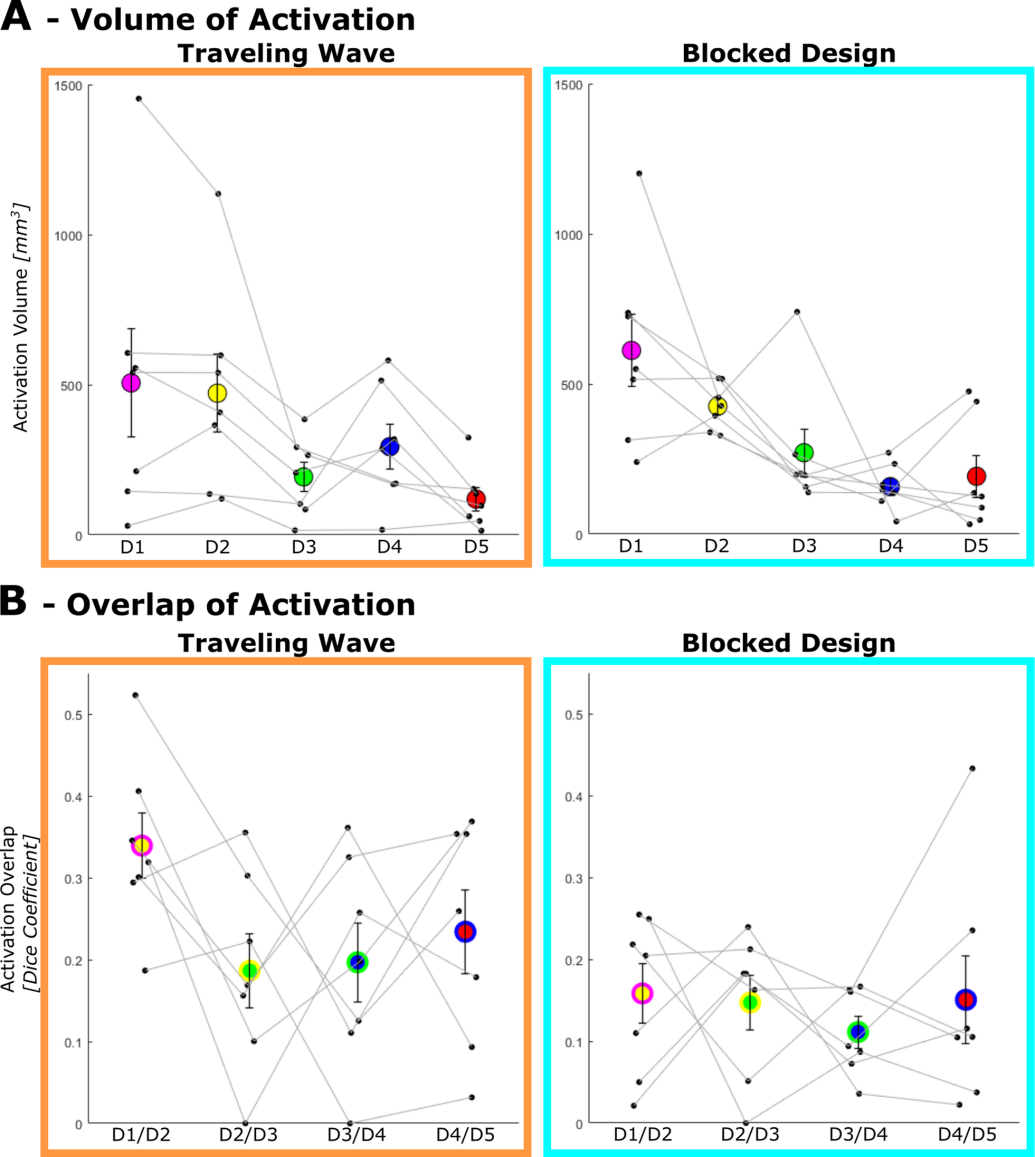
Activation volume and overlap of neighboring activation clusters extracted from the first session. **A** Average (large colored circles) and individual (small black circles) activation cluster volumes. D1 and D2 generally occupy the most space while the other three digits (D3, D4, and D5) have smaller activation volumes. This general pattern can be observed both for TW and BD. **B** Average (large two-colored circles) and individual (small black circles) measures of activation overlap between two neighboring activation clusters. There is a nonsignificant trend towards more overlap in the TW design as compared to the BD. Error bars represent the standard error of the mean

The size of the single digit BOLD-activation was calculated based on the 3D volume of the activation cluster (Fig. 5A). The average volume shows a decline across the digits, with D1 displaying the largest (TW: 506.1 ± 179.5 mm^3^; BD: 611.6 ± 121.1 mm^3^) volume of activation in both designs. D5 displayed the smallest volume of activation in the TW maps (119.0 ± 38.9 mm^3^) and D4 in the BD maps (157.9 ± 28.8 mm^3^). This was reflected in the rm-ANOVA in a significant DIGIT effect (*F*(1.2, 7.2) = 6.4, *p* = 0.035) of the volumes of activation. No evidence could be found for an effect of DESIGN, or an interaction effect DIGIT x DESIGN, indicating no evidence for differences in the volumes of activation between the two designs.

#### Overlap of activation between neighboring digits: dice coefficient

The overlap of activation between neighboring digits is quantified by the Dice coefficient (DC), which, in this case, describes the overlap of activation between two neighboring digits in relation to the entire activation clusters of the two digits, with higher values indicating larger overlap. The activation overlaps between neighboring digits reveals slightly higher average DC values in the TW design (minimum = 0.19 ± 0.05 (D2 + D3); maximum = 0.34 ± 0.04 (D1 + D2)) compared to BD (minimum = 0.11 ± 0.02 (D3 + D4); maximum = 0.16 ± 0.04 (D1 + D2)). In the rm-ANOVA of the DCs, this is moderately reflected as a trend towards a significant effect of DESIGN (*F*(1, 6) = 5.0, *p* = 0.067), which suggests higher specificity of the single digit activation in the BD approach, but could also be a consequence of the contrast of each digit predictor against the average of the other four digit predictors used solely in the analysis of the BD, biasing the analysis towards more digit specific voxels. No trend or significant effects concerning DIGIT PAIR, nor the interaction DESIGN x DIGIT PAIR is present, reflecting the similar DC of overlap of neighboring digits and their large variation.

### Retest reliability across first and second session

#### Stability of activation: dice coefficient of overlap across sessions

**Fig. 6:**
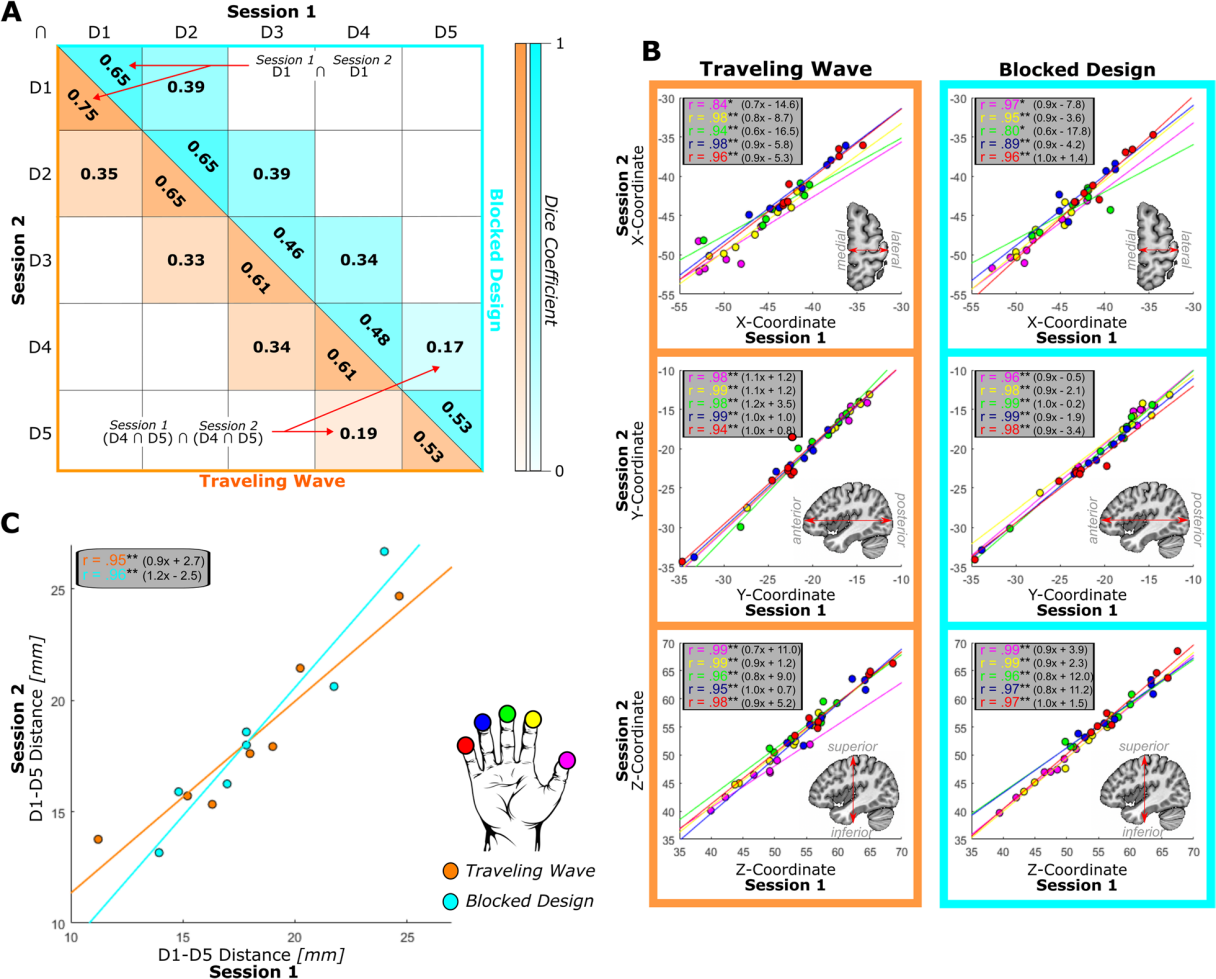
Retest reliability of location-based parameters. **A** Average Dice Coefficients (DC) describing the overlap of the activation cluster in the first session with the activation cluster of the second session, with higher values indicating more and lower values indicating less overlap. Values on the diagonal represent same-digit overlap: activation clusters for the same digit are compared between sessions. Values off the diagonal represent comparisons between the area of overlap of neighboring digits between sessions: the area that is common for the activation cluster of two digits in the first session is compared to the common area of the same two digits in the second session. The orange-colored side represents values for the TW design and the cyan colored side represents values for the BD. **B** All three axes (X, Y, and Z; rows) of each digit’s center of gravity (CoG) were correlated across the first and second session for both designs (columns). Data points represent individual data. All digits in all dimensions achieved significant correlation values indicating high stability of the location of the CoG. **C.** D1-D5 distances were correlated between the first and second session for both designs (colors). Data points represent individual data. Both designs achieved significant correlation values, indicating that the D1-D5 distance is a stable measure of the size of the somatosensory area of digit representation. Best fitting line described as (slope)x + (intercept).* p < 0.05; ** p < 0.001; after FDR correction

To describe the retest reliability of single digit activations the DC provides a general description of similarity. The spatial correspondence of a single digit activation between the first and the second session captures the overlap of the activation both in size and location across sessions. In the DC matrix (Fig. 6A), this is presented on the diagonal, showing a range of DC values for the inter-session overlap of the five digits. These DC values range between 0.46 and 0.75 implying an overlap of the single digit activation clusters between the two sessions of 46–75%, with no evidence for differences between the two mapping approaches (paired samples t-test on average values on the diagonal: *t*(6) = 1.3, *p* = 0.245).

The DC values off the diagonal indicate the spatial correspondence of the overlap of the four neighboring digit-pairs, by comparing the neighboring digit activation overlap of the first session, with the neighboring digit activation overlap of the second session. In this comparison the range of the DC values falls between 0.17 and 0.39, much lower compared to the DCs of the same digit activation, indicating that the activation overlap between neighboring digits is more variable across sessions compared to the overlap of the single digit activation clusters across sessions. Again, no evidence for a statistical difference between the DC of the spatial correspondence of activation overlap of neighboring digits was obtained (paired samples t-test on average values off the diagonal: *t*(6) = 1.0, *p* = 0.366).

As the DC represents a combined measure of stability of the location and the size of the activation, separate analyses focusing on the degree of variation in these two parameters between the two sessions were undertaken, aiming to discriminate the differential effect of location and volume of activation on the retest reliability.

#### Stability of single digit centers of gravity: correlations and absolute values across sessions

To quantify the retest reliability of the locations of single digit activation for both mapping approaches, we correlated each digits CoG X- (medial–lateral direction), Y- (anterior–posterior), and Z- (superior-inferior) coordinates of the first and the second session (Fig. 6B). The results exhibit very high correlation (TW: *M* = 0.96, Range: 0.84–0.99; BD: *M* = 0.96, Range: 0.80–0.99) values for all X- Y-, and Z-coordinates, which are all significantly larger than zero. Additionally, the best fitting lines are close to the line of equality with slopes close to 1 (TW: *M* = 0.91, Range = 0.6–1.2; BD: *M* = 0.95, Range = 0.6–1.0) and low intercepts (TW: *M* = 5.71, Range = 0.7–16.5; BD: *M* = 4.92, Range = 0.2–12.0). Together, these best fitting lines and high correlation values for all digits and in all three dimensions indicate a high stability and high retest reliability of the location of the single digit CoG for both the BD and TW design.

To quantify the differences in CoG locations across sessions, Euclidean distances of the digit CoGs between the first and second session were obtained for all participants (Table 4). Generally, the Euclidean distances confirmed the results of the correlation analysis that the CoGs were at similar positions in the two sessions. The individual subject difference in position of the single digit CoG across digits and designs was generally below the voxel size of 2 mm isotropic, with 20 out of 70 single digit activations (29%) showing a shift of more than one voxel size and only 7 cases (10%) a shift of more than two voxel sizes. The average Euclidean distances across participants revealed a deviation above 2 mm (one voxel shift) in the TW design for D1 and D5, but the average Euclidean distances across participants and digits were not different between TW (*M* = 1.95, *SD* = 1.56) and BD (*M* = 1.58, *SD* = 1.08) (*t*(34) = 1.3, *p* = 0.22), indicating no evidence for differences in retest reliability of the CoG location between the two mapping designs. These results underline that the position of the CoG is a reliable measure also on the level of single subjects.

**Table 4:**
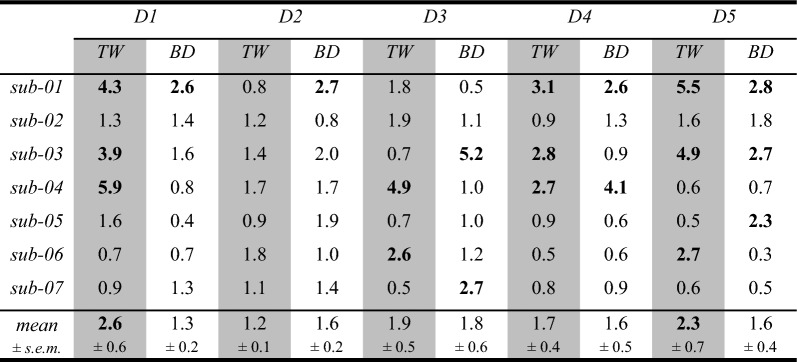
Euclidean distances (in mm) between the CoGs for the same digit in session 1 and session 2 for both TW (gray) and BD (white)

Values above 2 mm (i.e., size of one functional voxel) are marked in bold

#### Stability of D1-d5 distance: correlation across sessions

The retest reliability of the extent of the somatosensory hand area, estimated by the correlation of the first and second session geodesic distance between D1 and D5 CoG, also shows strong correlation values for both mapping approaches (TW: *r* = 0.84; BD: *r* = 0.88) (Fig. 6C). The best fitting lines for both designs are following the line of equality, indicating that the results of the measurements of the D1-D5 distance are very stable across sessions.

#### Stability of single digit volume of activation: correlation across sessions

**Fig. 7:**
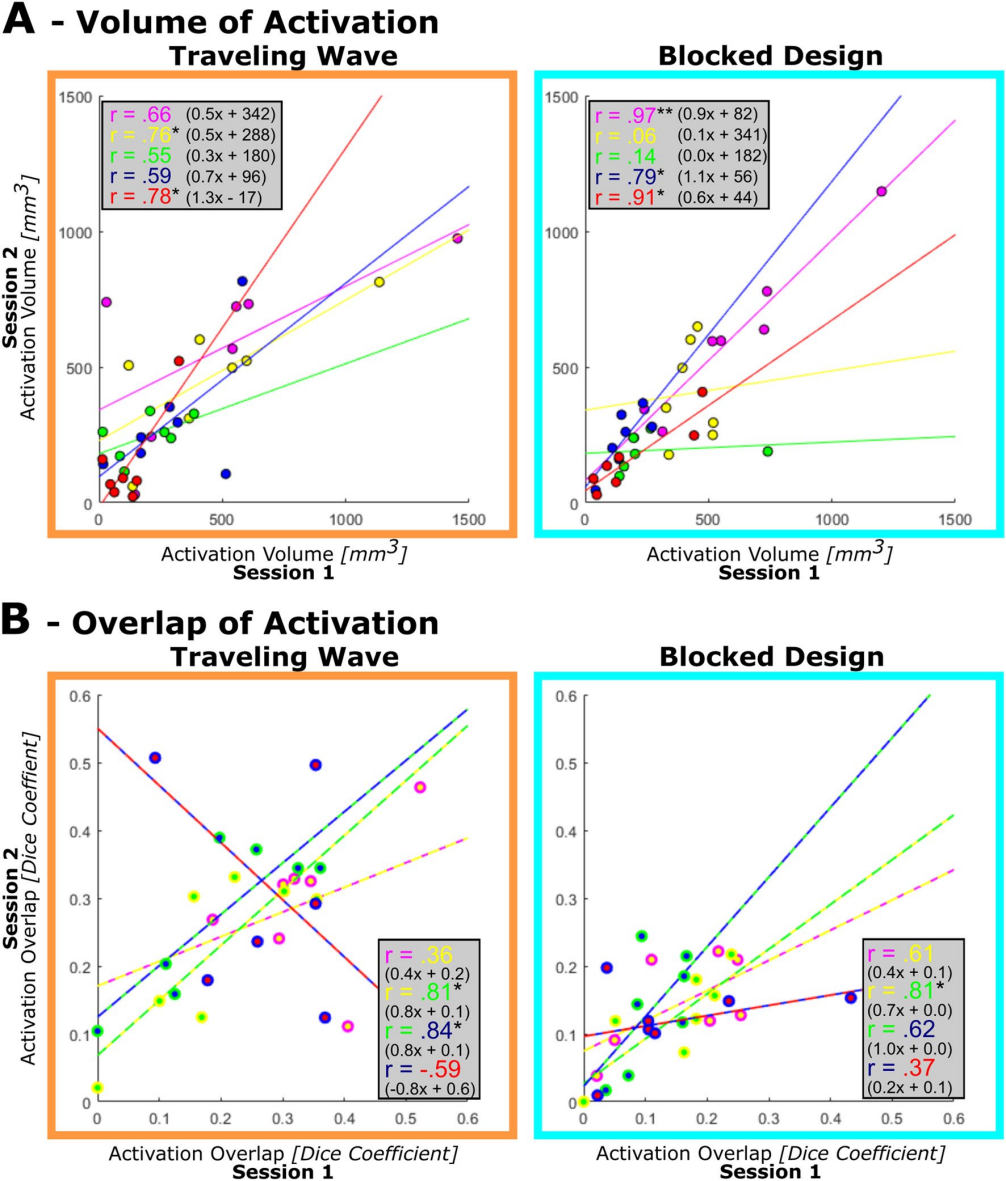
Retest reliability of the volume of the activation cluster and activation overlap. **A** The volume of the activation cluster from the first session was correlated with the second session for all five digits (color code identical to previous figures; D1 = magenta; D2 = yellow; D3 = green; D4 = blue; D5 = red). Data points represent individual participants. With a few exceptions, area of activation displayed medium to low correlation values indicating little stability across sessions. **B** The overlap of the activation of neighboring digits as quantified by the Dice coefficient was correlated between the first and second session for all four digit pairs (D1 + D2 = magenta/yellow; D2 + D3 = yellow/green; D3 + D4 = green/blue; D4 + D5 = blue/red). Most correlation values are low and non-significant, indicating little to no stability of the activation overlap across sessions. Best fitting line described as (slope)x + (intercept). * p < 0.05; ** p < 0.001; after FDR correction

The analysis of the retest reliability of the volume of each digit’s cortical activation across sessions revealed considerable variation in the correlation values of each digit’s size of activation between sessions (TW: *M* = 0.67, Range: 0.55–0.78; BD: M = 0.57*,* Range = 0.06–0.97) (Fig. 7A). In the TW approach, only the volume of activation of two digits, D2 and D5, are significantly correlated between sessions. In the BD, three digits, D1, D4, D5, reach significance, while the other two digits display low correlations (D2: r = 0.06 and D3: r = 0.14). In the case of D3, removing the influential point would result in a higher correlation (r = 0.89) but reduce the original variability of the complete dataset. Also, the best fitting lines do not resemble the line of equality in all cases. Especially D1, D2, and D3 (TW) as well as D2 and D3 (BD) diverge drastically. These results reveal that the volume of digit activations is not always stable across sessions for both mapping designs, indicating that there might be a difference in terms of reliability for the volume of activation of different digits.

#### Stability of neighboring digits’ overlap of activation: correlation across sessions

To assess the retest reliability of the overlap of neighboring digits, the DCs of the activation overlap of the four digit-pairs D1 + D2, D2 + D3, D3 + D4, and D4 + D5 were extracted for session 1 and 2 and then correlated (TW: *M* = 0.36, Range = −0.59 to 0.83; BD: *M* = 0.60, Range = 0.37–0.81) (Fig. 7B). For the TW, the DC values show a large range of values, which for only two digit-pairs resulted in significant correlations (D2 + D3: *r* = 0.81; D3 + D4: *r* = 0.84), one digit pair even produced a negative correlation (D4 + D5: *r* = −0.59). For the BD approach, the DCs are less scattered, but the correlations are low except for the D2 + D3 digit-pair (*r* = 0.81) which was significantly larger than zero. Again, in some cases, the best fitting lines do not resemble the line of equality, especially for the digit pairs D1 + D2 and D4 + D5 for both designs. These results suggest that the size of the activation overlap of most neighboring digit-pairs is not stable across sessions.

## Discussion

This preclinical translational fMRI study investigates the feasibility, validity, and retest reliability of traveling wave (TW) and blocked design (BD) digit mapping approaches adapted for clinical applications. Results confirm that complete and comprehensive digit maps can be obtained despite the constraints for both designs. The locations of the single digit activation CoGs display the commonly observed D5-D1 superior-inferior, medial–lateral and posterior-anterior succession along the central sulcus with high similarity between the designs. Area of digit activation varies between digits but is similar across designs, except for the larger overlap between neighboring digit activation for the TW compared to the BD design. The descriptions of the clinically relevant retest reliability show very high stability of the location of the digit activations between sessions for both designs, revealing that the location of digit representation can be reliably determined. This is contrasted by the area of activation and the overlap with neighboring digits activation exhibiting a considerable variability and only moderate stability across sessions making it a less reliable descriptor of the digit activation. In the following, these results are assessed in relation to existing data and future directions for optimization are discussed.

### Feasibility and validity of somatosensory digit mapping in a clinical adaptation

Both mapping designs, TW and BD, yielded significantly activated voxels in response to passive tactile stimulation of all five digits in both sessions for all participants, demonstrating the general feasibility of the adapted procedures to map the entire cortical hand representation in SI.

The considerably reduced spatial resolution of 2 mm isotropic (8 mm^3^), compared to the 1.5 mm isotropic (3.375 mm^3^) at 3 T or even 1.2 mm isotropic (1.7 mm^3^) at 7 T used in basic research studies, does limit the spatial specificity of the signal, and increases partial volume effects, considering the narrow width (< 2 mm) of SI [21, 45]. However, the findings that activation for all 5 digits was present in all participants shows that this disadvantage is counterbalanced by the larger voxel volume yielding a stronger signal and lower sensitivity to motion artifacts, which turns into an important benefit for the implementation in a clinical population.

The analysis of the location and area parameters of the single digit activation provide a valid description of the topography that is in line with the results from basic research studies: the known succession of the single digit centers of activation along the medial–lateral, superior-inferior and posterior-anterior trajectory of the posterior wall of the central sulcus [3, 38, 41, 46, 53], the comparable D1-D5 geodesic distances gauging the somatosensory hand area [29, 54], and the digit specific sizes of activation elicited by the tactile stimulation [41, 54] confirm the viability of the adaptations.

In addition to the general feasibility of the clinically adapted approaches in obtaining valid digit maps, it is important to emphasize that both mapping designs, TW and BD, yielded comparable results in almost all location- and area-based measures, despite the differences in stimulation and analysis, which speaks to their robustness. Furthermore, this may confirm a number of decisions made concerning the analysis and parameterization. First, the structural definition of BA 3b as the region of interest, although activation from BA3a or, more plausible, activation from BA1 cannot be excluded. Second, the removal of voxels significantly activated by three or more digits, potentially representing BOLD-activation originating from blood vessels and not gray matter, which could lead to false interpretation of the fMRI-based digit map under the specific conditions of lower magnetic fields at 3 T and lower spatial resolution [4, 46, 58]. Third, the limitation to one cluster per digit, following the description of the single digit representation within BA3b obtained with electrophysiology in monkeys [30, 43, 50]. Fourth, the selection of the center of gravity within this cluster to represent the location of the digit representation [64, 66]. Although, more studies and analyses, especially with patient populations, are needed to put these decisions further on trial.

The center of gravity approach was implemented in an automated approach to extract each digit’s activation cluster based on its size, the presence of the peak voxel, and in the case of diverging choices, also its location. This expert-based pipeline is, again, an adaptation to the clinical setting, with its strong need for automatized procedures, reducing the burden on health care practitioners, especially for rare procedures as this detailed fMRI analysis. A recent approach to create a standardized and automatized analysis pipeline for somatosensory digit mapping data based on the high spatial resolution fMRI and additional MR-angiography information [48] was informative, but not applicable for the clinical application due to its high spatial resolution and the need for an additional MR-angiography scan.

Though not formally confirmed, a noticeable difference between the two mapping designs is the larger and more variable overlap of the activation of neighboring digits in the TW compared to the BD. This, however, can most probably be ascribed to the different analysis approaches applied to the different stimulation schemes. The BD was analyzed with the standard GLM, and each digit predictor was contrasted against the average of the predictors for the remaining digits. For the phase-encoding cross-correlation analysis applied to the TW stimulation no contrast was applied. Moreover, the inclusion of two models (see *2. Methods and Materials*) for the cross-correlational analysis of each digit could have contributed to the increased activation overlap between neighboring fingers in the TW design. Differences in the adaptable FDR thresholding also have to be considered, as the level of thresholding influences the percentage of voxels being activated by two digits and consequently the extent of the overlap [4]. The specific analyses for this clinical adaptation were chosen to closely follow the approaches of published studies [34, 35, 54] to allow the comparison of the results to the results of these basic research studies.

Taken together the comparison of the TW and BD mapping results show each design’s power and capacity to map the somatosensory digit area even when taking into account possible limiting factors of a clinical setting. The two approaches achieve comparable results for the location of the single digit activation as well as for the volume of the activated area, which are analogous to basic research studies having the advantage of higher spatial resolution and higher field strengths.

### Retest reliability

The Dice coefficients, describing retest reliability by comparing the spatial correspondence of the single digit activation of the first and the second session, resulted in 51–75% overlap of the activation for both mapping designs. These values are in the same range as reported by Kolasinski and colleagues [34, 35] for their intra-subject single digit map reproducibility of an active mapping task applying the TW design. Despite the encouraging correspondence of this clinical adaptation with the results of the ultra high-field and high spatial resolution study, the actual values of the similarity between measurements are lower than hoped for, considering that the measurements were done in neurotypical volunteers. For the clinical implementation, with the additional challenge that the extent of the potential BOLD-activation changes associated with recovery and therapeutic interventions are not yet described, this retest variability has to be taken into account. Since the Dice coefficient, as a similarity measure, takes into account the location as well as the area of the digit activation, separate analyses of the retest reliability of the location and of the areas of the single digit activations were conducted to investigate the contribution of each of the components.

#### Retest reliability of location based measures

The subsequent correlational analyses of the single digit CoGs between the two sessions show high values with the correlation being very close to the line of equality for both designs, indicating a high retest reliability of the location of the single digit CoGs for all participants and both designs. These results converge with previous reports of the circumscribed across-session variability of the location of digit activations. Values of the between-session variance for somatosensory fMRI measurements at 3 T are not available, since location stability across sessions was, especially in the early digit mapping studies in humans, not explicitly investigated. This changed with ultra-high-field 7 T fMRI, providing higher spatial resolution to investigate not only reorganization, but potentially also digit map adaptations. There, data associated with retest reliability are occasionally reported as part of the general descriptions, showing a highly stable location of digit activation comparing different runs within the same session [61] as well as between sessions with longer intermediated time periods [41]. The actual reported average values of the distances between sessions are in the same range as the values in the present study ([41]: min = 1.7 ± 1.2 mm D1,max = 3.2 ± 3.1 mm for D2 | present study: min = 1.2 ± 0.1 mm D2; max = 2.6 ± 0.6 mm for D1) both demonstrating a displacement of an equivalent of 1–2 functional voxels. A confirmative retest reliability is reported in a 9.4 T, high spatial resolution MRI study in squirrel monkeys, assessing the limits for the description of reorganization/adaptation in BA3b across measurements weeks to month apart. The locations of the single digit activation peak voxels between sessions show an average variation of 0.5 ± 0.15 mm, which considering the in-plane spatial resolution of 625 × 625 µm^2^, also correspond to a displacement of the size of 1–2 functional voxels [71]. This variability of the single digit location across sessions by on average 1–2 voxels, even across different magnitudes of magnetic fields and spatial resolutions, demonstrates a proficient stability and retest reliability of the location of single digit activation, which is promisingly matched in the present pre-clinical implementation. Although, it has to be pointed out that, on the individual level, in 43% of the participants (3 out of 7), 40% of the digits (2 out of 5) showed a displacement of the CoG of three voxels (4.1—5.9 mm), which is due to the relatively coarse spatial resolution, already within the range of the distance to the CoG of the neighboring digit.

#### Retest reliability of volume based measures

Contrary to the location, the area of activation and the overlap between the areas of neighboring digit representations shows only medium to low correlation values and medium to large deviations from the line of equality. This indicates more pronounced variations in the elicited areas of the single digit activation across sessions and consequently a lower retest reliability.

Considerable within-subject variations of the extent of BOLD activations between sessions, despite using identical stimulation and measurement regimes, are not unusual and have been investigated and described in detail (e.g., [42]) providing the basis for the development of suitable preprocessing, analysis, and thresholding strategies (e.g., [60]). Concerning the specific topic of the extent of single digit activation across sessions, actual descriptions are, again, sparse and reveal a variety of sources. Martuzzi and colleagues [41] report a general decrease in the volume across all single digit activations in the second sessions, which the authors comprehensively explain by a drop in attention. In the present study, a counting task was associated with the tactile stimulation to assure a constant focus of attention on the stimulation across the sessions, and to prevent a general loss of signal across all single digit activations in the second session. A more general approach was taken by the already referred to high-field fMRI study in squirrel monkeys, in which the large variations in the size of the digit activation across sessions are attributed to changes in the signal to noise ratio. The comparison of different thresholding procedures revealed that a flexible, and stricter, thresholding approach resulted in much smaller areas of activation, which were more reproducible across sessions than the larger activations resulting from a fixed or a combined fixed/flexible thresholding approach [71]. The present study applies the False Discovery Rate (FDR) [2] as a flexible thresholding approach, which adapts to the general difference of BOLD signal between sessions, partially counterbalancing its effect on the number of activated voxels. But even under the condition of FDR threshold adapting from measurement to measurement, there is a considerable difference in area variability between the single digits causing digit specific decrements in the retest reliability. One of the possibilities to reduce this variability is to increase the threshold (see [71]) to select voxels with strong enough activation that the BOLD signal fluctuations lose relevance [42]. But this approach, although very successful in increasing the retest reliability of the area of BOLD-activation, would in the extreme case approximate the area around the peak voxel and thereby converge with the location retest reliability. More strict thresholds would also reduce the specificity for the potential and probably subtle changes in the extent of the activation reflecting changes in the underlying neuronal digit representation, as would clinically be expected associated the adaptation within the digit map in response to recovery and/or treatment interventions changes in the digits and hand usage. Further studies are needed to balance out this conflict between reliability and specificity, both being important for the description of the layout of the digit map as well as for the detection of changes within this layout.

#### Retest reliability of overlap based measures

The retest reliability of the overlap of neighboring digit activation shows an, at least equally large, variation and low correlation across the two sessions as the retest reliability of the area of activation. This is also reflected in the low Dice coefficients showing an average correspondence of only 36% between the neighboring digit overlaps in the two sessions, also indicating larger fluctuations of the overlap of neighboring digit activation. The only other study mentioning the retest reliability of the overlap of digit activation across sessions depicts the within subject fluctuation of Dice coefficients of the digit activation overlap between sessions and show that the between subject variation of the overlap between digits and sessions is larger than the within subject variation [34, 35]. Enhanced intra-subject reliability could be obtained, as discussed with the area of activation, by more conservative thresholds, reducing the area of activation, and consequently reducing the overlap between neighboring digit activations. Again, additional high spatial resolution fMRI studies at ultra-high fields are needed to clarify the different factors influencing the reported overlap of activation of neighboring digits [4, 34, 35, 58] as well as addressing the general question of how the layout of the individual neuronal digit representations can reliably be captured, to provide a basis for an adaptation into the specifics of clinically applicable mapping procedures.

### Limitations of the study

Vibrotactile mapping procedures such as the ones employed in this study only capture a subset of the somatosensory system. It is currently not described how the tactile and the other subsystems of somatosensation, and their interactions influence different aspects of stroke related motor recovery. However, given the various limitations posed by the MRI environment and the clinical setting, vibrotactile stimulation evoked cortical activation can, at present, be a beneficial proxy for probing specific aspects of somatosensory cortical functionality.

Despite the clinical focus of this implementation of digit mapping procedures, this research was not performed with patients but with young neurotypical participants. This limitation to the general validity of the approach had to be taken into account for two reasons, namely to be able to adequately describe the feasibility as well as the retest reliability of the clinical adaptation. To specify the feasibility, two different mapping approaches were compared. Consequently, multiple measurements had to be performed within a session, which would not have been reasonable nor acceptable for older adults or patients. The description of retest reliability on the other hand, required two of these multiple measurement sessions with the additional prerequisite that no alterations in the topographical organization of the digit map are expected to occur during the intermediate time, which would be an unrealistic prospect in patients. There are, evidently, clear limitations in the transfer of the results of the clinical adaptation of the procedures, if they are tested in neurotypical young adults only. The generalization of findings from neurotypical participants to patient populations must be approached with caution, since even fundamental parameters can differ, such as a slightly altered hemodynamic response function in stroke patients [6], calling for an adapted fMRI analysis in these patients.

Likewise, the sample size of seven participants is rather small, though comparable to other studies investigating the reliability of somatosensory digit mapping procedures ([35]: n = 9; [41]: n = 10,[61]: n = 6). That sample size does not always matter, can be read in the insightful neuroimaging analysis study of Marek and colleagues [40] establishing that big samples of thousands of individuals can be necessary to perform specific studies, e.g., brain wide associations, but the authors also do not hesitate to stress the importance of small sample within-subject studies for clinical care. Moreover, as the aim of the current study was to investigate the feasibility and reliability of somatosensory mapping for a clinical context, the focus was on the individual, rather than the group level. When the obtained digit map parameters are expected to be used for diagnostics or prognosis of treatment outcome, the description of the variability on the level of the individual has to be included in the description of the reliability.

## Conclusion

This study shows that the fMRI-based mapping of the somatosensory digit representations in SI can, even on the individual level, be feasible, when taking certain limitations of the clinical setting into consideration. The valid description of the general topography within BA3b of the somatosensory cortex exhibiting the succession of the single digit representation is an important prerequisite to determine potential reorganization of the digit maps in clinical conditions. The additional description of the retest reliability, showing a high stability of the location of the digit representation over time, compared to a lower replicability of the extent of the digit representation and its overlap with neighboring digit representations. While no major differences in terms of feasibility nor retest reliability were found between the two mapping designs, the more conventional statistical analysis procedure might favor the blocked design for clinical application. This is a promising first step towards the clinical assessment of somatosensory cortex reorganization due to trauma and digit map adaptability due to interventions.

## Data Availability

The data that support the findings of this study are available on reasonable request from the corresponding authors, RS or TS.
